# When and How Do Emotional Intelligence and Flourishing Protect against Suicide Risk in Adolescent Bullying Victims?

**DOI:** 10.3390/ijerph16122114

**Published:** 2019-06-14

**Authors:** Lourdes Rey, Sergio Mérida-López, Nicolás Sánchez-Álvarez, Natalio Extremera

**Affiliations:** 1 Department of Personality, Evaluation and Psychological Treatment, Faculty of Psychology, University of Málaga, 29071 Málaga, Spain; 2 Department of Social Psychology, Social Work, Social Anthropology and East Asian Studies, Faculty of Psychology, University of Málaga, 29071 Málaga, Spain; sergioml@uma.es (S.M.-L.); nextremera@uma.es (N.E.); 3 Department of Developmental and Educational Psychology, Faculty of Psychology, University of Málaga, 29071 Málaga, Spain; nsa@uma.es

**Keywords:** suicidal ideation, depressive symptoms, flourishing, emotional intelligence, adolescence, bullying victimization

## Abstract

This study contributes to current knowledge on the protective role of emotional intelligence and flourishing in cases of suicide risk (namely depressive symptoms and suicidal ideation) in a sample of adolescent victims of traditional bullying. The proposed model tested the mediator role of flourishing in the relationship between emotional intelligence (EI) and suicide risk together with the moderating effect of EI in the relationship between low flourishing and increased suicide risk. Considering an initial sample of 1847 adolescents (52.5% female), a subsample of 494 pure bullying victims (61.3% female) took part in this research. The main results showed EI to be linked to decreased suicide risk through levels of flourishing. Moreover, EI buffered the relationship between low flourishing and the associated suicide risk. Victimized adolescents with both low levels of EI and of flourishing reported higher levels of suicide risk than their counterparts with high EI levels. This suggests the protective role of EI of both predicting higher flourishing and reducing the likelihood of suicide risk among victimized adolescents with low levels of flourishing. Finally, the practical implications of these novel findings regarding the role of EI and flourishing in the prevention of suicide risk among victimized adolescents are discussed.

## 1. Introduction

In recent decades, schools worldwide are facing a major public health problem that is related to aggressive behaviors among peers [1]. School bullying, generally defined as repeated exposure over time to face-to-face aggressions in which one person or a group intentionally cause pain against a victim who cannot easily defend him- or herself [2], is regarded as a public health problem requiring serious attention at multiple levels [3]. This call-for-action is a response to the adverse consequences bullying posits on adolescents’ adjustment, health, and well-being.

Previous research studies have shown that the negative impact victimization has on adolescents’ health is stable over time [4]. A meta-analytic review has provided consistent evidence on a causal association between bullying victimization and mental health problems, including higher suicidal thoughts and behaviors [5]. According to the main findings of a systematic review, the number of the experienced adversities or negative life events seemed to have a positive dose-response relation with youth suicidality [6]. This finding is critical as it suggests risk accumulation regarding the risk of suicide among adolescents. Another internalizing indicator of poor mental health (depression) is consistently shown as a critical consequence after experiencing a negative life event [7] and as an outcome of victimization [8]. Because bullying is causally related to suicide [5], this major concern requires critical attention from researchers, institutions, and administrations worldwide so that effective, preventive efforts can be made.

### 1.1. Suicide Risk among Bullying Victims

As described above, there is a mounting body of research showing that adolescent victimization impairs adolescents’ health, adjustment, and well-being [5,9]. Most importantly, suicide constitutes a critical outcome that can be predicted by adolescents’ bullying victimization [10]. Considering the widely-established continuum process, with suicidal ideation at one end of the continuum and death by suicide on the other end [11,12], a growing number of studies have focused on suicidal ideation as a critical proximal factor predicting suicide in the context of bullying [11,13]. Nonetheless, empirical evidence advocates the inclusion of depressive symptoms as a distal predictor of suicide risk [12,14]. Indeed, depression is highly involved in suicide [15,16]. Thus, an increasing number of research studies have used both indicators of internalizing problems (i.e., depressive symptoms and suicidal ideation instruments) for assessing suicide risk among samples of adolescents and college students [17,18]. Accordingly, we assessed suicide risk associated with victimization by means of examining depressive symptoms and suicidal ideation.

The intensity of the consequences of bullying on adjustment and health depends on the role played in this phenomenon. Indeed, bullying victims are more likely to develop worse psychological health states, with higher levels of internalizing problems, namely depressive symptoms and suicidal ideation, than other involved adolescents as the bystanders [11,18]. It has been shown that the risk of suicide is three to five times higher for victims than for uninvolved adolescents [19]. Furthermore, bullying victims also display high levels of negative emotions such as fear, sadness, loneliness, shame, embarrassment, rumination, and anger [20,21], which, in turn, would increase the likelihood of becoming involved in aggressive behavior such as turning into perpetrators [22]. Therefore, there is a need to consider not only general samples of adolescents who may be involved in school bullying but to specifically focus on particular roles at greater psychosocial risk, such as victims [23].

### 1.2. Emotional Intelligence as a Psychological Resource Predicting Suicide Risk in the Context of Bullying Victimization

Despite the importance paid to risk factors that might be associated with suicide risk among victimized adolescents, previous studies have shown that the associations between victimization and internalizing problems are not absolute, which suggests that not all victimized adolescents are at the same risk for poor mental health [8]. A recent line of research has begun to consider the potential benefits of addressing a more positive psychological perspective to increase well-being in order to prevent suicide risk [24]. The impairing process that bullying victimization represents for adolescents’ health leads to a need for evidence on the individual factors that might promote well-being and protect bullying victims against suicide risk. Among these personal psychological resources, the emotional intelligence (EI) construct is a dimension capturing individuals’ ability to deal with affective information [25]. The ability-based EI approach defines this construct as a set of emotional skills for processing information about emotions and emotion-relevant stimuli, and to use this information as a guide to thinking and behavior in order to promote emotional and intellectual growth [25].

In recent decades, there has been an accumulative review showing that EI is consistently related to indicators of mental health [26] and subjective well-being [27]. Moreover, a recent review has provided consistent empirical evidence on the associations between EI and suicide risk in diverse populations, including adolescents [28]. With respect to school bullying, low EI explains involvement in aggressive behaviors [29] and bullying towards peers [30]. Moreover, the EI construct has gained growing interest in the field of school health and well-being because of its correlations with health and well-being associated with victimization in traditional bullying [13,31]. In sum, EI is suggested to be an individual resource that might protect adolescents against the impact of bullying and that attenuates the deleterious emotional impact of victimization on emotional well-being [31].

### 1.3. Emotional Intelligence, Flourishing, and Suicide Risk among Victimized Adolescents

Despite the growing evidence on the relationship between EI and decreased suicide risk among bullying victims, literature on the positive mechanisms linking these variables is still limited. According to the need of adopting a positive psychology approach in the prevention of suicide risk [24], there is a dimension that may contribute to explaining the decreased suicide risk rates associated with bullying victimization, reflecting positive mental well-being, namely flourishing [32,33]. According to Keyes’ framework, this variable is conceptualized as a combination of emotional, psychological, and social well-being [33]. Through a comprehensive lens, flourishing examines both hedonic (i.e., positive emotions and satisfaction toward one’s life) and eudaimonic (positive functioning regarding intrapersonal and interpersonal levels in one’s life) well-being. Additionally, this construct reflects positive social functioning (i.e., social acceptance and integration). Thus, flourishing constitutes a novel approach that integrates classic components of well-being and is, thus, regarded as a promising background for understanding well-being [34].

Following the proposed model of flourishing [33,35], researchers place mental well-being in a continuum with ends fluctuating between low and high flourishing. Individuals who have high flourishing scores report psychological, emotional, and social well-being. For example, high flourishing has been linked to higher emotional stability [36] and physical health [37], more positive social relationships [38] and better psychosocial functioning [32]. Conversely, flourishing is negatively related to loneliness [39] and may explain levels of poor mental health and suicide [40]. In fact, a study with a large sample of adolescents provided data on the high prevalence of depressive symptoms among youth whose individual scorings were low in flourishing [41]. Furthermore, a research with a large sample of college students conducted by Keyes and colleagues [40] showed that flourishing did contribute to explaining mental disorders and suicidal behaviors. Thus, levels of flourishing may significantly predict changes in suicide risk among victimized adolescents.

**Figure 1:**
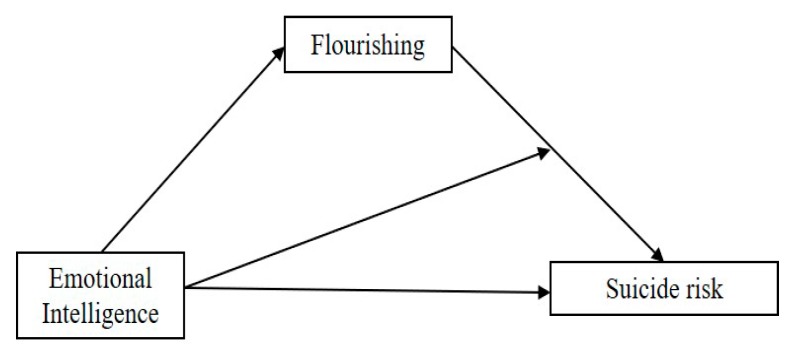
Illustration of the proposed conceptual model.

Although the relationship between EI and flourishing has not yet been explicitly addressed, prior meta-analytic research has suggested that EI is associated with diverse well-being outcomes [27]. Moreover, regulation of emotions, a key dimension of EI, has been found to be a significant predictor of flourishing levels [42]. Since emotional abilities have been suggested as a protective factor against suicide [28,43], it is plausible that EI may predict decreased suicide risk among victimized adolescents through levels of flourishing. Relatedly, earlier evidence suggests that EI is a moderator in the relationship between low well-being outcomes and increased suicide risk [44]. Additionally, EI is related to involvement in roles of school bullying [30,45], mitigating the effects of victimization on suicidal thoughts and behaviors [46]. Based on these findings, our proposed moderated-mediation model is displayed in Figure 1.

### 1.4. Contributions, Objectives and Hypotheses of Our Research

Despite the great amount of attention paid to incidences of traditional bullying and its various consequences in different geographic areas, settings, and cultures [47], there are several avenues to be addressed in the current literature on school bullying. Considering a more positive psychology perspective regarding school health and well-being appears to be crucial so that more effective positive prevention strategies can be developed [24].

This study aims to contribute to the current literature in the field of school health and well-being in three ways. First, we investigate EI and flourishing as antecedents of suicide risk in a sample of traditional bullying victims. Research on EI, flourishing, and suicide risk among bullying victims would allow us to better understand how emotional responses to victimization are managed in terms of related psychological, emotional, and social well-being and subsequent suicide risk. Second, this study contributes to the field of school bullying by means of specifically targeting a sample of victims in order to provide useful data to prevent consequences of bullying for a group that is at particular risk [11]. Finally, there is a need to consider research showing that the results of bullying interventions within schools are discrete in terms of efficacy [48]. Thus, our results may provide useful evidence to be used in designing more effective strategies to reduce the impact of victimization on suicide risk [24,48].

Based upon prior literature, this study examines, through a moderated-mediation model (1), whether the association between EI and suicide risk (including both depressive symptoms and suicidal ideation) is mediated by flourishing, and (2) whether the indirect association between EI and suicide risk via flourishing depends on EI levels (see Figure 1). A better knowledge of the predictive role of EI on suicide risk through flourishing (mediation analyses) would allow this field of research to design future intervention programs including a wider perspective of psychological well-being that would include the significance that psychological, social, and emotional well-being have on adolescence development [41]. Additionally, testing the buffering role of EI on the relationship between low flourishing levels and high suicide risk (moderation analyses) might significantly contribute to the line of protective factors towards suicide risk associated with bullying victimization [43,49].

Based on prior literature, we propose the following specific hypotheses:

**Hypothesis** **1a.** **(H1a).** *The relationship between EI and depressive symptoms as a suicide risk is mediated by flourishing.*

**Hypothesis** **1b.** **(H1b).** *The relationship between EI and suicidal ideation as a suicide risk is mediated by flourishing.*

**Hypothesis** **2a.** **(H2a).** *The indirect effect of EI on depressive symptoms as a suicide risk through flourishing is moderated by EI levels.*

**Hypothesis** **2b.** **(H2b).** *The indirect effect of EI on suicidal ideation as a suicide risk through flourishing is moderated by EI levels.*

## 2. Materials and Methods

### 2.1. Participants

A convenience sample of adolescents from nine educative center from southern Spain participated in this cross-sectional study (*N* = 1847). Their ages ranged from 12 to 17 years old (M = 14.55, SD = 1.67). A subsample of actual bullying victims was selected according to the criteria used by Elipe et al. [50]. Thus, the final sample comprised 494 adolescent bullying victims (61.3% female). The sample was mostly Spanish (91.7%). The missing values were imputed using the imputation algorithm of expectation-maximization with SPSS 22 (SPSS Inc., Chicago, IL, USA) [51]. This procedure was followed for those participants with a minimum of 80% of the scales completed. Those subjects who did not meet the minimum were discounted to take part in the study sample.

### 2.2. Measures

#### 2.2.1. Emotional Intelligence

The Wong and Law Emotional Intelligence Scale (WLEIS) [52] is a self-report measure that contains four dimensions of EI (16 items): appraisal of one’s own emotions, appraisal of others’ emotions, use of emotion, and regulation of emotion. Students were asked to respond to a 7-point Likert scale ranging from 1 (“totally disagree”) to 7 (“totally agree”). We used the overall score in our analyses as we were interested in the role of EI as a whole [53]. There are several reasons supporting the use of this self-report measure of EI. First, this scale is based on Mayer and Salovey’s (1997) conceptualization of EI [25], and so it is regarded as a reliable method to access to emotional-affective processes. Second, this test has been satisfactorily used in earlier studies on adolescents’ health and adjustment [54,55]. Finally, the WLEIS was used considering practical reasons as it is relatively short and easy to administer. In this study, we used the Spanish version which has shown adequate validity and reliability in Spanish contexts (α = 0.91 and Ω = 0.94) [56]. In our sample, the Cronbach’s alpha for the total EI score was 0.84.

#### 2.2.2. Flourishing

The Flourishing Scale (FS) [57] is a measure of the core aspects of social-psychological functioning. This scale has a single factor structure and adequate reliability in different samples. The FS consists of eight items phrased in a positive manner that describe several aspects of human functioning, such as positive relationships, feelings of competence, and having meaning and purpose in life. Each participating adolescent was asked to answer on a 1 (“strong disagreement”) to 7 (“strong agreement”) Likert-type scale. In this study we used the validated Spanish version because of it has an adequate stability, reliability, and criterion validity (Ω = 0.89) [58]. The Cronbach’s alpha in this sample was 0.87.

#### 2.2.3. Suicide Risk

To assess suicide risk in adolescents, we used two different measures (i.e., depression and suicidal ideation) given the association between these variables [59,60,61]:

Depression was measured with the subscale of the Depression, Anxiety, and Stress Scale (DASS-21) [62]. This self-report measure assesses psychological symptoms of depression, anxiety, and stress with a 4-point Likert scale (“0 = did not apply to me at all” to “3 = applied to me very much, or most of the time”). In this study we used the Spanish version of the DASS-21, which has shown good reliability (α = 0.84) [63], focusing on the items assessing depressive symptoms. In our sample, the Cronbach’s alpha was 0.88.

Suicidal ideation was assessed using the Frequency of Suicidal Ideation Inventory (FSII) [64]. The FSII consists of five items that describe the frequency of suicidal thoughts over the past year on a 5-point Likert scale, from 1 (never) to 5 (every day). High scores indicate excessive suicidal thoughts. In this study, we used the Spanish version of FSII because of the high internal consistency and reliability (α = 0.89 and Ω = 0.92) [65]. In our sample, the Cronbach’s alpha was 0.89.

### 2.3. Procedure

Head teachers of the schools were responsible for reporting and consulting with the adolescents’ families about the study, explaining that by completing the questionnaires adolescents were providing informed consent for us to use this data in the present research. Then, head teachers provided written informed consent for the conduct of the study in the school. The data collection was conducted in classrooms during a 1 h lesson, with the presence of one of the researchers and one of the school’s teachers. Instructions were given in classrooms with guarantees of the participants’ voluntariness and anonymity. The study was carried out in accordance with the ethical principles for psychological research involving human subjects and was approved by the Research Ethics Committee of the University of Málaga (62-2016-H).

### 2.4. Data Analyses

Statistical analyses were carried out using SPSS software to calculate means, standard deviations, reliabilities for the measured variables, and correlation coefficients. Moderated mediation analyses were conducted using SPSS PROCESS macro v3.0, model 74, (SPSS Inc., Chicago, IL, USA) [66]. This model integrates a mediation relationship with a moderation effect of the predictor on the second path of the mediator model and it is recommended as it calculates overall and conditional indirect effects for these models of moderated mediation via the counterfactual approach [67]. Indeed, previous studies have followed this procedure to test similar moderated mediation models [68,69] as it generates accurate and reliable results [70]. Consistent with the proposed model (see Figure 1), overall EI was the predictor as well as the moderator for the relationship between the mediator (i.e., flourishing) and the dependent variable (i.e., suicidal ideation and depressive symptoms). Gender and age were statistical controls. In accordance with guidelines, we used 5000 bootstrap resamples and calculated 95% confidence intervals.

## 3. Results

### 3.1. Descriptive Statistics

Descriptive statistics (means, standard deviations, reliabilities, and correlations) are provided in Table 1.

### 3.2. Moderated Mediation Models

The results of the moderated mediation model for depressive symptoms and suicidal ideation as outcomes are displayed in Table 2.

#### 3.2.1. Results Regarding Depressive Symptoms

The first model showed that EI (*β* = −0.09, *p* = 0.007; Lower Limit of the 95% Confidence Interval or LLCI/Upper Limit of the 95% Confidence Interval or ULCI = −0.16/−0.02) and flourishing predicted depressive symptoms (*β* = −0.02, *p* < 0.001; LLCI/ULCI = −0.03/−0.02), which suggests that EI has an indirect relationship with depressive symptoms that is mediated by flourishing (mediation hypotheses: H1a). Moderated mediation analyses indicated that EI interacts with flourishing to predict levels of depressive symptoms (*β* = 0.01, *p* < 0.001; LLCI/ULCI = 0.01/0.02). The interaction term explained a 2% unique variance in depressive symptoms (∆R^2^ = 0.02). At low levels of overall EI (Coefficient = −3.562), the conditional, unstandardized, and indirect effect of flourishing on depressive symptom was –0.06 (bootstrapped confidence intervals LLCI/ULCI = −0.08/−0.04). At high levels of EI (Coefficient = 1.378), the indirect effect of flourishing on depression was lower (Coefficient = −0.01, bootstrapped confidence intervals LLCI/ULCI = −0.02/−0.01). This finding was confirmed by the moderated mediation index and the associated bias-corrected bootstrap confidence intervals (Index = –0.02, LLCI/ULCI = −0.03/−0.02), indicating that the conditional, indirect effects estimated at low and high levels of the moderator were different from each other (moderation hypotheses: H2a).

#### 3.2.2. Results Regarding Suicidal Ideation

In the first model, EI predicted flourishing (*β* = 4.60, *p* < 0.001; LLCI/ULCI = 3.81/5.39). Suicidal ideation was predicted by flourishing (*β* = –0.17, *p* < 0.001; LLCI/ULCI = −0.21/−0.12) and so EI has an indirect relationship with suicidal ideation that is mediated by flourishing (mediation hypotheses: H1b). Moreover, EI interacted with flourishing to predict suicidal ideation (*β* = 0.05, *p* < 0.001; LLCI/ULCI = 0.02/0.09). The interaction term explained a 1.6% unique variance in suicidal ideation (∆R^2^ = 0.016). While at low levels of overall EI (Coefficient = −3.562), the conditional, unstandardized, and indirect effect of flourishing on suicidal ideation was −0.38 (bootstrapped confidence intervals LLCI/ULCI = −0.50/−0.27). At high levels of EI (Coefficient = 1.378), the indirect effect of flourishing on suicidal ideation was lower (Coefficient = −0.09, bootstrapped confidence intervals LLCI/ULCI = −0.16/−0.02). This finding was confirmed by the moderated mediation index and the associated bias-corrected bootstrap confidence intervals (Index = −0.17, LLCI/ULCI = −0.22/−0.13), indicating that the conditional indirect effects estimated at low and high levels of the moderator were different from each other (moderation hypotheses: H2b).

**Figure 2:**
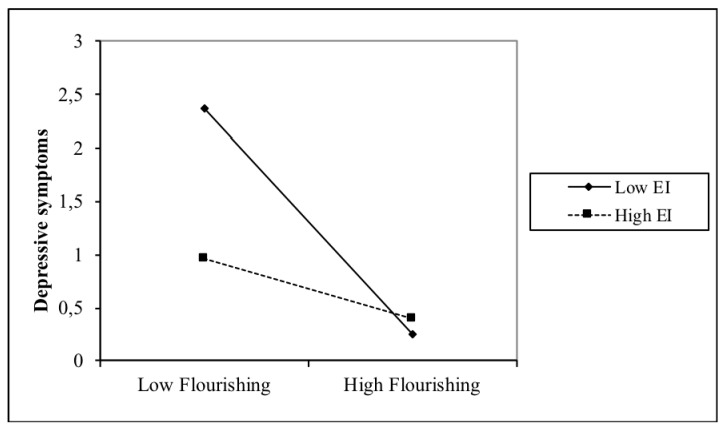
Interaction between EI and flourishing with regards to depressive symptoms.

Figure 2 illustrates the interaction between EI and flourishing with depressive symptoms. Flourishing and depressive symptoms were associated at low EI levels (*β* = −0.03, *t*(509) = −10.222, *p* < 0.001), but this association was weaker at high EI levels (*β* = −0.01, *t*(509) = −3.862, *p* < 0.001). Post hoc analyses showed that the slopes of the two lines were different (*t* = 2.96, *p* = 0.003).

**Figure 3:**
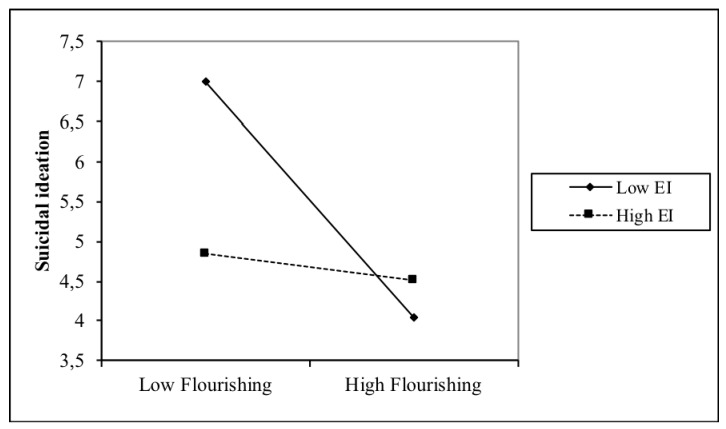
Interaction between EI and flourishing with regards to suicidal ideation.

Figure 3 illustrates the interaction between EI and flourishing with suicidal ideation. Flourishing and suicidal ideation were associated at low EI levels (*β* = −0.23, *t*(509) = −9.801, *p* < 0.001), but the association was weaker at high EI levels (*β* = −0.12, *t*(509) = −3.945, *p* < 0.001). Post hoc analyses showed that the slopes of the two lines were different (*t* = 2.86, *p* = 0.004).

## 4. Discussion

The aim of this study was to contribute to the research literature of school health and well-being by (1) examining direct and indirect relationships between EI, flourishing, and suicide risk in a sample of traditional bullying victims; and (2) exploring the role of EI as a buffering factor in the relationship between flourishing and suicide risk. The results of this study support the mediation path of EI on suicide risk, namely depressive symptoms and suicidal ideation through flourishing (H1a and H1b), and the moderating role of EI on the relationship between flourishing and suicide risk (H2a and H2b).

Firstly, and in line with prior research [11,13,71], adolescent bullying victims showed a negative relationship between EI and depressive symptoms and suicidal ideation. While the association between EI and suicide risk was negative [28], its link with flourishing was positive [27]. Additionally, there was a significant negative association between flourishing and adolescents’ suicide risk, which is consistent with past studies [40,41]. Further, there was an indirect relationship between EI and suicide risk via flourishing, which makes its potential mediator role stand out. Thus, as expected, H1a and H1b were supported, which suggests that flourishing has a key role in the protective effect of EI on suicide risk in adolescent bullying victims [35,40].

Secondly, EI had a moderating effect on the associations between flourishing and suicide risk, thereby confirming H2a (depressive symptoms) and H2b (suicidal ideation). The negative association between flourishing and suicidal ideation was exacerbated when adolescent bullying victims had low EI levels, especially at low levels of flourishing. However, when bullying victims reported high EI levels, even when reporting low flourishing levels, the suicidal risk became smaller than it was for these adolescents’ low-EI counterparts. Nonetheless, when they reported higher levels of flourishing, the bullying victims’ reported EI levels, regardless of how different, became less important. Regarding the relationship between flourishing and depressive symptoms, a similar pattern was found. When adolescents reported low levels of flourishing, their high reported EI did make a difference regarding the levels of depressive symptoms. These results are in line with research on bullying, which has suggested that children and young people with different positive personal resources will respond differently after becoming a bullying victim [46,72]. So, it appears that emotional abilities might act as relevant, positive resources, and they might potentially explain the subsequent suicide risk [31,46,73]. When adolescent bullying victims have reported low degrees of psychological, emotional, and social well-being (flourishing), those who have scored higher EI levels are less vulnerable to experience suicide risk after being victimized. Hence, our findings suggest that when adolescents report high levels of flourishing, having EI has a less significant effect on their experience of being victimized.

Although this finding is in line with prior research that suggests that the links between lower levels of well-being and mental health indicators depend on the levels of positive personal resources [18], further work could profitably examine the role of flourishing and the contribution of EI to variance in suicide risk.

### 4.1. Strengths and Limitations

This study adds evidence to the literature on positive resources and factors explaining school health and well-being. Two major strengths of the present study are the use of a specific sample of adolescents: traditional bullying victims and the inclusion of a recent mental well-being construct, namely flourishing, which combines hedonic and eudaimonic well-being. To the best of our knowledge, this is the first study to explore flourishing in the context of bullying victimization and its relationship with EI to predict suicide risk in a sample of adolescent victims.

Some limitations must also be noted in our study. First, the cross-sectional design makes it difficult to establish dynamic relationships. Although our preliminary findings are theory-driven and accord with previous studies showing that EI is related to positive mental health, well-being, and decreased suicide risk among victimized adolescents [13,27,31], it remains impossible to rule out the potential influences among variables. The preliminary results of the current study suggest the fruitfulness of using positive strategies to reduce the impact of bullying victimization on suicide risk [24,48]. However, longitudinal designs are required to provide a more rigorous test for our hypothesized model so that conclusions about causality in studied relationships are drawn. Likewise, future studies could valuably assess the longitudinal effects of adolescents’ EI on their adjustment regarding their role within bullying contexts [30]. Second, our results are also limited by the use of questionnaires for data collecting. Although self-report instruments are inexpensive and practical, there are potential limitations regarding common method biases. We followed recommendations for reducing the likelihood of this issue [74]. For instance, we relied on measures with adequate construct validity and used a format of responses with no right or wrong answers. Nonetheless, future studies should use both self-reports and other forms of data, such as interviews, performance-based EI tests, or teacher rating data.

### 4.2. Practical Implications and Future Research

Despite the aforementioned limitations, this research has provided new evidence for the proposed, moderated mediation model and suggested further avenues for designing future and more effective intervention programs based on positive perspectives. On the one hand, it seems fruitful to put the most effort into increasing the levels of flourishing to reduce maladjustment after victimizations. As flourishing is a relatively recent mental well-being construct [35,40], its comprehensive lens may contribute to the current knowledge on adolescents’ well-being from a positive perspective, considering emotional, psychological, and social dimensions. If Seligman’s prediction that by the year 2051, 51% of the worldwide population will be flourishing in a personal way, highlighting the proud inheritance of positive psychology [75], more research is needed to find more modifiable antecedents of flourishing [76].

On the other hand, based on our study, EI has been a key variable-like antecedent, not only of flourishing but also of suicide risk among bullying victims. Thus, EI could be considered a relevant, positive-personal resource for coping with bullying victimization [77]. Additionally, since we found strong relationships between EI and flourishing, it would also be interesting to examine how other variables might contribute to achieving more well-being or even how other factors (i.e., gender) might explain differences in adjustment when bullying or other stressful life events occur. For example, some researchers have shown that girls typically report a greater tendency to be attentive to moods and higher interpersonal perception compared with boys [45,78]. Furthermore, women tend to be more vulnerable to the impact of stressful life events [79], with male adolescents reporting less psychological symptoms than females [80]. In addition to this, scientific literature shows differences related to gender in emotions, subjective well-being, and positive psychological functioning [81,82]. Therefore, an important avenue for future studies would be to examine the potential role of gender as an underlying mechanism that could interact in the relationship between EI dimensions, well-being indicators, and suicide risk in adolescent bullying victims.

Regarding practical implications, prevention and intervention programs should take into consideration not only anti-bullying policies and curriculum-based activities, but also training on EI to manage school-related stressors and the negative emotions associated with them. EI training can contribute to improve EI and conflict resolution skills together with impulse control and anger management [77]. Since EI is significantly related to prosocial goals and behaviors, this training could be beneficial not only in terms of the prevention of aggression and bullying problems among adolescents but also in terms of improving the quality of the relationships among peers. Finally, our findings suggested low EI as a risk factor for decreased flourishing and increased suicide risk among victims of bullying. Thus, EI training among adolescents should be regarded as a promising avenue for the enhancement of positive mental health within school settings. This training may be focused among adolescents with low EI levels, as previous results have shown the greatest effectiveness of socioemotional programs for this group at greater risk for maladjustment [77].

## Figures and Tables

**Table 1:** Means, standard deviations, reliabilities, and correlations of the variables of interest.

Variables	1	2	3	M	SD	α
1. EI	-			4.74	0.92	0.84
2. Flourishing	0.48 **	-		5.32	1.15	0.87
3. Suicidal ideation	−0.27 **	−0.44 **	-	1.84	0.88	0.89
4. Depressive symptoms	−0.33 **	−0.48 **	0.59 **	0.87	0.71	0.88

Note: M= Mean; SD= Standard Deviation; **α** = Cronbach’s alpha; EI = Emotional intelligence; ** *p* < 0.01.

**Table 2:** Tested models with depressive symptoms and suicidal ideation as outcomes.

Variables	B	SE b	R^2^	95% CI
Depressive symptoms			0.24 ***	
Constant	−0.44	0.25		(−0.94, 0.06)
Gender	0.15 **	0.05		(0.03, 0.26)
Age	0.07 ***	0.01		(0.03, 0.10)
EI	−0.09 **	0.03		(−0.16, −0.02)
Flourishing	−0.02 ***	0.01		(−0.03, −0.02)
EI × flourishing	0.01 ***	0.03		(0.01, 0.01)
Suicidal ideation			0.24 ***	
Constant	6.23 ***	1.64		(3.01, 9.45)
Gender	1.35 ***	0.36		(0.63, 2.07)
Age	0.39	0.10		(−0.16, 0.24)
EI	−0.36	0.21		(−0.79, 0.06)
Flourishing	−0.17 ***	0.02		(−0.21, −0.12)
EI × flourishing	0.05 **	0.01		(0.02, 0.09)

Note: B = Beta; SE b = Standard error; 95% CI = 95% Confidence Intervals; EI = Emotional Intelligence; ** *p* < 0.01; *** *p* < 0.001.
